# Polymorphism rs547984 on human chromosome 1q43 is not associated with primary open angle glaucoma in a Saudi cohort

**DOI:** 10.1186/s12952-017-0077-0

**Published:** 2017-06-26

**Authors:** Taif A. Azad, Nikhil B. Edward, Altaf A. Kondkar, Hatem Kalantan, Saleh Altuwaijri, Tahira Sultan, Faisal A. Al-Mobarak, Saleh A. Al-Obeidan, Khaled K. Abu-Amero

**Affiliations:** 1 Glaucoma Research Chair, Department of Ophthalmology, College of Medicine, King Saud University, Riyadh, 11411 Saudi Arabia; 2 Case Western Reserve University, Ohio, USA; 3 SAAD Research & Development Center, Clinical Research Lab., SAAD Specialist Hospital, P.O. Box 30353, Al Khobar, 31952 Saudi Arabia; 4 Qassim University, Buraydah, Saudi Arabia; 5 Department of Ophthalmology and Visual Sciences, University of Illinois at Chicago, Chicago, IL USA; 6 Ophthalmic Genetics Laboratory, Department of Ophthalmology, College of Medicine, King Saud University, P.O. Box 245, Riyadh, 11411 Saudi Arabia

**Keywords:** Poag, rs547984, Saudi, *ZP4*

## Abstract

**Background:**

To investigate the association between polymorphism rs547984, located in close proximity to the Zona Pellucida Glycoprotein 4 (*ZP4*) gene on human chromosome 1q43 and primary open angle glaucoma (POAG).

**Method:**

Polymorphism rs547984 was genotyped using Taq-Man® assay in 185 subjects comprising of 90 unrelated POAG cases and 95 controls of Saudi origin.

**Results:**

Association analysis between cases and controls revealed no significant genotype distribution under additive (*p* = 0.356), dominant (*p* = 0.517) and recessive (*p* = 0.309) models. Besides, the allele frequency distribution was also found to be non-significant (*p* = 0.70). The minor “A” allele frequency was found to be 0.49 and 0.50 among POAG cases and controls, respectively. In addition, specific clinical indices used to assess severity of glaucoma such as intraocular pressure (IOP), cup/disc ratio and number of anti-glaucoma medication also did not show any significant genotype distribution in POAG cases.

**Conclusion:**

Polymorphism rs547984 is neither associated with any clinical indices important for POAG such as IOP and cup/disc ratio nor is a risk factor for POAG in the Saudi cohort.

## Background

Primary open angle glaucoma (POAG) is the second most common form of glaucoma in Saudi Arabia and a leading cause of irreversible blindness worldwide [1]. POAG is known to have a genetic component with two- to four-fold risk of POAG in first-degree relatives as compared to the general population. However, due to the polygenic nature of POAG with genetically complex and multifactorial inheritance its exact genetic etiology is still unknown [2].

Recently, using population-based genome wide association study (GWAS), several investigators have identified a number of variants in multiple loci/genes to be associated with POAG and related quantitative traits that may contribute to the development and/or progression of the disease in various ethnic groups [3]. Nakano et al. described the first GWAS in in a group of Japanese POAG patients and identified 3 genetic loci consisting of six single nucleotide polymorphisms (SNPs) to be associated with POAG. Of these, 4 intergenic SNPs including rs547984, located on human chromosome 1q43 flanking the Zona Pellucida Glycoprotein 4 (*ZP4*) gene were found to be in strong linkage [4]. *ZP4* gene is involved in functions related to fertilization and preimplantation development and thus far SNPs and/or mutations in this gene were reported in association with ovarian diseases [5] and POAG [4]. However, subsequent studies have failed to replicate this association between rs547984 and POAG in a South Indian [6], Afro-Caribbean [7], Japanese [8] and Korean [9] population. Besides, the functional relevance of the SNP or the gene to POAG development is not known.

Despite a high prevalence of POAG in Saudi population the role of genetic polymorphisms in the development of the disease is still unclear [3]. Our previous attempts to identify genetic associations between POAG and polymorphisms in genes including caveolin (*CAV1/CAV2*) [10], atonal homolog 7 (*ATOH7*) [11], cyclin-dependent kinase inhibitor 2B (*CDKN2B*) [12], and transmembrane and coiled-coil domain 1(*TMCO1*) [13] did not yield any positive results indicating that the genetic cause for POAG in patients of Saudi origin may be different than those from European descent. In addition, there are no reports of association of polymorphisms in *ZP4* gene in the middle-eastern POAG patients. With the aim to identify a genetic link and provide further validation of this POAG-associated variant in a different ethnic group, we investigated whether SNP rs547984 is associated with POAG in the middle-eastern cohort of Saudi Arabia.

## Methods

### Study design and population

The study adhered to the tenets of the Declaration of Helsinki and all participants signed an informed consent. The study was approved by the College of Medicine ethical committee (approval number # 08-657). To perform a case-control study Saudi patients with a clinically confirmed diagnosis of POAG and a matching group of glaucoma free healthy controls were recruited into the study at King Abdul-Aziz University Hospital (KAUH) in Riyadh, Saudi Arabia. A total of 185 subjects comprised of 90 POAG patients and 95 controls of Saudi origin were included in this study. POAG patients satisfied the following strict clinical criteria for inclusion: i) appearance of the disc or retinal nerve fibre layer e.g. thinning or notching of disc rim, progressive changes, nerve fibre layer defect; ii) the presence of characteristic abnormalities in the visual field (e.g. arcuate scotoma, nasal step, paracentral scotoma, generalized depression) in the absence of other causes or explanation; iii) age greater than 20 years at the time of recruitment and iv) open anterior chamber angles bilaterally on gonioscopy. Exclusion criteria included evidence of secondary glaucoma, e.g. pigmentary glaucoma, uveitic, pseudoexfoliation, or any other form of secondary glaucoma, and history of steroid use or ocular trauma. Healthy controls free from glaucoma by examination were recruited. Inclusion criteria for controls were age > 20 years, normal intraocular pressure (IOP) [IOP < 21 mmHg without any anti-glaucoma medication], open angles on gonioscopy, and normal optic disc on examination.

### DNA preparation

DNA samples were obtained from peripheral blood (7 mL) collected in EDTA tubes from all participating individuals. Extraction was performed using the illustra blood genomicPrep Mini Spin kit (GE Healthcare, Buckinghamshire, UK) and stored at −20 °C in aliquots until further use. Quantification of extracted DNA was performed using a NanoDrop ND-2000c spectrophotometer (Thermo Scientific, Wilmington, DE, USA).

### Genotyping of rs547984 at Chr.1: 237,933,586 on GRCh38

Subjects were genotyped for polymorphism rs547984 using the TaqMan® SNP Genotyping Assay (Applied Biosystems Inc., Foster City, CA, USA) on ABI 7500 Real-Time PCR System (Applied Biosystems) as described previously [14]. For detection of rs547984 polymorphisms, assay IDs: C_8859642_10 was used. A 25 μL PCR reaction consisted of 1X TaqMan® Genotyping Master Mix (Applied Biosystems), 1X SNP Genotyping Assay Mix and 20 ng DNA. Each 96-well plate included two no template controls. Real-time PCR was performed on an ABI 7500 using the recommended conditions consisting of incubation at 95 °C for 10 min, followed by 40 cycles, denaturation at 92 °C for 15 s and annealing/extension at 60 °C for 1 min. The VIC® and 6-carboxy-fluorescein (FAM) fluorescence levels of the PCR products were measured at 60 °C for 1 min. Analysis of fluorescence using the automated 2-color allele discrimination software on ABI 7500 showed clear discrimination of both the genotypes on a two-dimensional graph.

### Statistical analysis

Data was presented as mean ± standard deviation for continuous variables and as numerical counts and percentages for nominal variables. Hardy-Weinberg Equilibrium (HWE) deviation was tested by Pearson’s Chi^2^ test. The Chi^2^ test was used to detect any association between the different genetic profiles (Fishers 2 × 3 Exact test when applicable). Risk was expressed as odds ratio (OR) and confidence interval (CI) level was set to 95%. Normality testing of continuous variables was done using Kolmogorov Smirnov test. Independent samples *t*-test was used to investigate whether there was any significant difference between cases and controls in terms of continuous variables. One-way ANOVA and Kruskal-Wallis tests were used to detect the mean difference across the three genotypes within POAG group. A *p* value less than 0.05 was considered statistically significant. All the analysis was done using SPSS version 22 (IBM Inc. Chicago, IIinois, USA).

## Results

No significant age and gender distribution was seen between POAG cases and controls. In addition, except for the family history of glaucoma (*p* = 0.012), systemic co-morbidities, smoking habit and glaucoma specific clinical indices were found to be similar between the two study groups (Table 1).

**Table 1:** Demographic and clinical characteristics of POAG cases and controls

Variables	Controls (*n* = 95) No. (%)	Cases (*n* = 90) No. (%)	*p* value^a^
Demographic Characteristics			
Age in years, mean (±SD)	57.1 (13.5)	60.7 (12.3)	0.061^b^
Male	69 (72.6)	55 (61.1)	0.096
Female	26 (27.3)	35 (38.8)	-
Systemic Diseases			
Diabetes mellitus	45 (47.3)	49 (54.4)	0.334
Coronary artery disease	6 (6.3)	6 (66.6)	0.920
Hypertension	44 (46.3)	47 (52.2)	0.420
Hypercholesterolemia	9 (9.4)	14 (15.5)	0.210
Health Awareness/Behavior			
Family history of glaucoma	4 (4.2)	14 (15.5)	0.010
Smoking	41 (43.1)	34 (37.7)	0.458

^a^Chi^2^ test, ^b^ *t*-test

The distribution of rs547984 polymorphism did not deviate significantly from the HWE (*p* > 0.05). Association analysis between cases and controls revealed no significant genotype distribution under additive (Chi^2^ = 2.07, df = 2, *p* = 0.356), dominant (*p* = 0.517) and recessive models (*p* = 0.309). Besides, the allele frequency distribution was also found to be non-significant (OR = 1.08; 95% CI = 0.72 – 1.62; *p* = 0.70). The minor “A” allele frequency was found to be 0.49 and 0.50 among POAG cases and controls, respectively (Table 2).

**Table 2:** Association analysis of SNP rs547984 with allele frequency and genotype distribution in POAG patients and controls

SNP (Nearest gene)	rs547984 (*ZP4*)				
Allelic analysis	Controls (*n* = 95) No. (%)	POAG (*n* = 90) No. (%)	Odds ratio	95% confidence interval	*p* value^a^
C	95 (50.0)	92 (51.1)	1	Reference	-
A^b^	95 (50.0)	88 (48.9)	1.08	0.72 – 1.62	0.708
HWE P	0.60	0.13	-	-	-
Genotype and Model analysis					
C/C	25 (26.3)	20 (22.2)	1	Reference	-
C/A	45 (47.3)	52 (57.7)	0.69	0.34 – 1.41	0.310
A/A	25 (26.3)	18 (20.0)	1.11	0.47 – 2.58	0.806
Additive (Trend)	-	-	-	-	0.356^c^
Dominant	-	-	1.25	0.63 – 2.45	0.517
Recessive	-	-	0.70	0.35 – 1.39	0.309

^a^Chi^2^ test, ^b^Risk variant, HWE P – Hardy-Weinberg equilibrium *p* value, ^c^Fisher exact test

We further evaluated the effect of genotypes on different demographic and clinical variables in the POAG group. Age (*p* = 0.674) and gender (*p* = 0.553) were found to be non-significant. Similarly, family history, smoking and systemic diseases also showed no significant distribution. More importantly, specific clinical indices used to assess severity of glaucoma such as IOP, cup/disc ratio and number of anti-glaucoma medication too were found to be non-statistically significant between the genotype groups (Table 3).

**Table 3:** Effect of rs547984 genotypes on demographic and clinical characteristics within PAOG group

Characteristics	Genotypes			*p* value^a^
	C/C (*n* = 20) No. (%)	C/A (*n* = 52) No. (%)	A/A (*n* = 18) No. (%)	
DEMOGRAPHIC				
Age in years, Mean (SD)	58.6 (13.8)	61.5 (12.1)	60.5 (11.6)	0.674^b^
Male	13 (65.0)	33 (63.4)	9 (50.0)	0.553
Female	7 (35.0)	19 (57.5)	9 (50.0)	-
MEDICAL HISTORY				
Family history of glaucoma	4 (20.0)	9 (17.3)	1 (5.5)	0.408
Smoking	10 (50.0)	18 (34.6)	6 (33.3)	0.440
Diabetes mellitus	13 (65.0)	27 (51.9)	9 (50.0)	0.556
Hypertension	9 (45.0)	28 (53.8)	10 (55.5)	0.758
Coronary artery disease	2 (10.0)	4 (76.9)	0 (0.0)	0.421
Hypercholesterolemia	5 (25.0)	9 (17.3)	0 (0.0)	0.091
GLAUCOMA INDICES				
Intraocular pressure in mmHg, mean (SD)	33.9 (6.1)	35.2 (8.3)	31.3 (5.7)	0.206^c^
Cup/disc ratio	0.69 (0.19)	0.74 (0.16)	0.65 (0.23)	0.285^c^
No. of anti-glaucoma medications	2.8 (0.6)	2.8 (0.8)	2.4 (0.6)	0.065^c^

^a^Chi^2^ test, ^b^One-way ANOVA, ^c^Kruskal Wallis test

In addition, to ascertain the effects of age, gender and genotype on the likelihood of having POAG a binary logistic regression analysis was performed. However, none of these factors could significantly explain the likelihood of POAG in this cohort (Table 4).

**Table 4:** Effect of age, sex and genotype on disease outcome by binary logistic regression analysis

Variables	Odds ratio	95% confidence interval	*p* value
Age	1.02	0.99 – 1.04	0.090
Sex^a^	0.59	0.32 – 1.11	0.101
Genotype^b^	-	-	0.425
C/A	1.33	0.648 – 2.763	0.431
A/A	0.84	0.355 – 1.982	0.689

^a^Female as reference, ^b^C/C as reference

## Discussion

This study investigated the association between SNP rs547984, which is in close proximity to *ZP4* gene on chromosome 1q43, and POAG patients of Saudi origin. Previously this SNP was reported to be associated with POAG in a group of Japanese patients (*p* = 0.00006, OR = 1.34, 95% CI = 1.16 – 1.54) [4]. However, the authors did not offer any explanation on how SNP rs547984 contributes to POAG-pathogenesis. Subsequent studies have failed to establish a link between this SNP and POAG [6–8]. In our study, the genotype and allele frequencies detected in POAG patients were comparable to those in controls and thus were insignificant. This is similar to previous investigations which reported no association between this SNP and POAG or one of its clinical indices [6–8]. A comparison of minor allele frequency distribution in different population is shown in Table 5. A minor allele frequency of 0.50 in Saudi controls was comparatively similar to the Japanese (0.46) [8] and Korean (0.484) population [9], but higher than the Indian (0.363) [6] and Afro-Caribbean (0.305) [7] population.

**Table 5:** Comparison of rs547984 minor allele (A) frequency distribution among different ethnic group

Population	MAF^a^ Controls	MAF POAG	*p* value	Reference
Japanese	0.460	0.560	0.00033	[4]
India	0.363	0.417	0.437	[6]
Afro-Caribbean	0.305	0.317	0.657	[7]
Korea	0.484	0.540	0.093	[9]
Saudi	0.500	0.489	0.708	This Study

^a^Minor allele frequency

Association analysis of the genotype effect on clinical parameters also did not provide any significant link. Smoking and other systemic diseases did not show any significant difference. More importantly, glaucoma specific indices such as IOP, cup/disc ratio and number of anti-glaucoma medication also showed no statistically significant difference between genotype groups. Therefore, this SNP, independently or in relation to other clinical indices, does not have any effect on POAG development. However, the findings of this study require cautious interpretation since the study is limited by its sample size, performed in specific ethnicity and the role of other polymorphisms in this gene cannot be ruled out.

## Conclusion

Our study was unable to replicate the previously reported association for variant rs547984 flanking the *ZP4* gene with POAG and glaucoma specific indices such as IOP and cup/disc ratio indicating that this SNP is not a risk factor for POAG in the Saudi cohort. Furthermore, in the light of the present findings and the lack of association reported among other ethnic groups it appears that this polymorphism may not have a role in POAG development.

## Abbreviations

CI: Confidence interval
GWAS: Genome-wide association study
HWE: Hardy-Weinberg Equilibrium
IOP: Intraocular pressure
OR: Odds ratio
POAG: Primary open angle glaucoma
SNP: Single nucleotide polymorphism
*ZP4*: Zona Pellucida Glycoprotein 4
